# Interpersonal emotion regulation in daily life: a comparison of individuals with and without depressive disorders

**DOI:** 10.3389/fpsyg.2026.1773706

**Published:** 2026-09-30

**Authors:** Pham Khanh Linh Vu, Karen Ollrogge, Luisa Daldrup, Stephan Köhler

**Affiliations:** 1 Department of Psychology, Clinical Psychology and Psychotherapy, University of Potsdam, Potsdam, Germany; 2 Department of Psychiatry and Psychotherapy, Charité—Universitätsmedizin Berlin, Corporate Member of Freie Universität Berlin and Humboldt-Universität zu Berlin, Berlin, Germany

**Keywords:** alexithymia, childhood maltreatment, cognitive reappraisal, depression, ecological momentary assessment (EMA), emotion regulation, social sharing, suppression

## Abstract

**Background:**

Emotion dysregulation is strongly linked to depressive disorders, yet distinctions between interpersonal and intrapersonal forms remain underexplored in daily life. This study examines emotion dysregulation in depression during close interpersonal interactions, comparing individuals with depression and healthy controls (HC), and exploring associations with alexithymia and childhood maltreatment.

**Methods:**

Participants (18 HC, 17 with depression) reported three emotion regulation (ER) strategies (cognitive reappraisal, social sharing, suppression) during interpersonal interactions over 2 weeks (3 prompts/day). Group (depression vs. HC) was the independent variable, and ER strategies the dependent variables. Alexithymia, childhood maltreatment, and depressive symptoms were assessed at baseline. Data were analyzed using two-level hierarchical linear modeling.

**Results:**

No group differences in ER strategy use were observed, and depressive symptom severity was not associated with strategy intensity. Post-hoc analyses showed that alexithymia was positively associated with depressive severity across the full sample and negatively associated with social sharing within the depressed group. Cognitive reappraisal showed heterogeneous associations with childhood maltreatment subdomains.

**Conclusion:**

Interpersonal emotion dysregulation did not differentiate depressed from healthy individuals. Given the small sample size, these null findings should be interpreted cautiously. Nevertheless, alexithymia and childhood maltreatment appear relevant for ER in close relationships, highlighting their importance in depression research.

## 1 Introduction

Globally, an estimated 4.4% of the population suffers from depressive disorders [World Health Organization (WHO), 2023], with a worldwide increase of 18.4% between 2005 and 2015 (Vos et al., 2016). Depressive disorders, characterized by persistent low mood and diminished interest or pleasure (Falkai et al., 2025), are closely linked to emotion dysregulation, a core process that remains insufficiently understood. Research suggests that individuals with depression rely more on maladaptive emotion regulation (ER) strategies and less on adaptive ones compared to healthy controls (HC) (Visted et al., 2018; Petschauer et al., 2022). However, prior work has not consistently distinguished between intrapersonal and interpersonal forms of ER, with interpersonal regulation remaining comparatively underexplored. In this study, social sharing is conceptualized as an inherently interpersonal strategy, whereas reappraisal and suppression are considered intrapersonal processes assessed within interpersonal contexts. This study aims to contribute to the growing body of research on interpersonal ER in depressive disorders. In addition, dispositional factors such as childhood maltreatment and alexithymia are considered, as both have been linked to altered ER and increased vulnerability to depression.

### 1.1 Emotion regulation: from adaptive—maladaptive dichotomy to context dependence

ER refers to the processes by which individuals influence their emotional experiences, including when emotions arise and how they are expressed (Gross, 1998). Early frameworks classified ER strategies as either adaptive or maladaptive based on their general associations with psychopathology (Aldao et al., 2010; Aldao and Nolen-Hoeksema, 2012). However, recent evidence indicates that their effectiveness is highly context-dependent (Aldao and Nolen-Hoeksema, 2012), particularly in interpersonal situations. Research in non-clinical samples shows that the use of social sharing and expressive suppression varies with interpersonal context: when individuals perceive their environment as high in social support, they engage in more sharing and less suppression, whereas lower perceived support reduces sharing and increases suppression (Pauw et al., 2022). Similarly, following the 1989 California earthquake, social sharing initially had beneficial effects, but became less effective over time as interpersonal receptivity decreased, illustrating how “social constraint” can alter its adaptiveness (Pennebaker and Harber, 1993). Building on this evidence, the present study examines ER strategies in real-world interpersonal contexts, focusing on cognitive reappraisal, suppression, and social sharing due to their relevance for both general functioning and depressive disorders (Troy et al., 2013; Schraub et al., 2011; Morawetz et al., 2021; Wagner et al., 2015).

### 1.2 Contextual influences on reappraisal, suppression, and social sharing in depression

Existing research indicates that cognitive reappraisal, suppression, and social sharing exert context-dependent effects on negative emotions and depressive symptoms, although most evidence stems from non-clinical samples (Troy et al., 2013; Khatibi et al., 2021). Cognitive reappraisal, reframing situations to alter emotional impact (Gross and Muñoz, 1995), has been shown to reduce depressive symptoms under uncontrollable stress but may exacerbate them in controllable situations in healthy populations (Troy et al., 2013), suggesting that its adaptiveness is context-dependent. Suppression, defined as the inhibition of emotional expression (Gross and Muñoz, 1995), has been associated with increased depressive symptoms in a non-clinical sample (Gross and John, 2003). However, in a non-clinical sample with high intolerance of uncertainty, suppression appeared linked to lower contamination-related anxiety (Khatibi et al., 2021), suggesting that in high-risk contexts expressive suppression may serve as an adaptive function by facilitating more effective regulation of worry (Khatibi et al., 2021). Social sharing, defined as verbalizing emotions to obtain support (Rimé, 2009), occurs less frequently in depressive disorders than in HC, potentially reducing perceived social support and contributing to symptom persistence (Lakey and Cronin, 2008). Its effectiveness depends on relationship closeness, with support from close others being more beneficial than from strangers in healthy populations (Morawetz et al., 2021; Wagner et al., 2015). Segrin and Flora (1998) further showed that individuals with depression display more negative language with friends than with strangers, whereas healthy individuals do not, highlighting the relevance of close interpersonal contexts. Paul et al. (2023) found that, in a healthy population, the presence of close others (e.g., family, friends) modulates the adaptiveness of ER: greater use of suppression in the presence (vs. absence) of close others was associated with higher depressive symptoms. Additionally, a decrease in reappraisal following increased presence of close others was linked to improved mental health (Paul et al., 2023).

Extending these findings, the present study examines ER in individuals with depressive disorders compared to HC within close interpersonal relationships. We hypothesize that in social situations with close partners, individuals with depressive disorders will exhibit greater use of reappraisal and suppression and reduced social sharing, compared to HC, reflecting maladaptive ER in interpersonal contexts. Higher depression severity is expected to correlate with greater reappraisal and suppression and lower social sharing. To capture these strategies in real-world settings, Ecological Momentary Assessment (EMA) will be employed, a method that captures relevant phenomena in an unbiased and immediate way within natural environments (Shiffman et al., 2008).

### 1.3 The role of childhood maltreatment and alexithymia in emotion regulation

ER is central to depressive disorders, yet less is known which factors may shape this pattern. Childhood maltreatment has been linked to the onset and maintenance of depression (Hovens et al., 2012; Chapman et al., 2004; Nanni et al., 2012), potentially via disrupted ER (Abravanel and Sinha, 2015; Yin et al., 2022). Similarly, alexithymia, characterized by difficulties in identifying and expressing emotions (Hogeveen and Grafman, 2021), is frequently elevated in depression (Sagar et al., 2021) and associated with reduced adaptive and increased maladaptive ER strategies (Preece et al., 2023). Based on theoretical considerations, both factors are expected to relate to less adaptive and more maladaptive ER in interpersonal contexts, as well as higher depressive symptoms. However, their specific interplay in close interpersonal settings remains insufficiently understood and may vary across associations. Therefore, these relationships are examined on an exploratory basis in individuals with depressive disorders.

## 2 Methods

### 2.1 Participants

From April to July 2023, 46 individuals were recruited via the Department of Psychiatry and Psychotherapy at Charité and social media. Inclusion criteria were: (a) age 18–65, (b) German fluency at C1 or higher, and (c) Android smartphone ownership. Exclusion criteria, assessed with the Mini-DIPS interview (Margraf and Cwik, 2020), included active substance addiction (*n* = 1), psychotic symptoms (*n* = 2), acute suicidality (*n* = 1), or benzodiazepine use (*n* = 1). Depressive participants met ICD-10 criteria for a current depressive episode or persistent dysthymia; HC had no psychiatric diagnoses. Participants with compliance below 40% were excluded to ensure comparable response rates, as including all intention-to-treat participants showed no significant group differences. The final sample included 35 participants: 18 HC and 17 depressive subjects (17 female, 18 male; Age: *M*_Healthy_ = 43.22, *SD* = 13.05; *M*_Depressive_ = 42.53, *SD* = 10.79). Most were single (*n* = 22, 62.85%). Reports of physical violence (*n* = 9, 52.94%), sexual violence (*n* = 2, 11.76%), and other highly distressing events (*n* = 15, 88.23%) were exclusive to depressive participants, as assessed with the Childhood Trauma Questionnaire. Among them, 15 had chronic/recurrent (*n* = 8, 47%) or episodic depression (*n* = 7, 41%), and 2 (12%) had a current episode without formal diagnosis, assessed using the Mini-DIPS. At survey start, all depressive participants had Beck-Depression-Inventory II (BDI-II) > 13 (*M* = 26.47, *SD* = 11.51), whereas HC averaged *M* = 5.22 (*SD* = 4.50).

### 2.2 Procedure

The assessment procedure consisted of a baseline screening, a diagnostic interview, self-report questionnaires, and a subsequent EMA phase. After providing consent, participants completed a baseline assessment on REDCap, including demographics and current mental state (e.g., depressive symptoms). They then underwent a Mini-DIPS diagnostic interview. The Mini-DIPS is a structured clinical interview based on ICD-10 criteria, administered by trained Bachelor’s students in psychology either in person at Charité Campus Mitte or via telephone (≈ 60 min). It was used to assign participants to the depressive or HC group, independent of prior diagnoses. Assessors were not blinded to recruitment status. Eligible participants then completed online questionnaires assessing childhood maltreatment, alexithymia, and depressive symptoms (≈30 min). All participants subsequently took part in a 14-day EMA survey via the movisensXS app (movisens GmbH, n.d.) on their smartphones. Participants received up to €30 for achieving at least 65% compliance.

### 2.3 Measures

#### 2.3.1 Baseline measures

Depression severity was measured with the 21-item BDI-II (Beck et al., 1996; German version: Hautzinger et al., 2023), rated on a 0–3 scale and classified as none/negligible (0–13), mild (14–19), moderate (20–28), or severe (≥29) (Beck et al., 1996; Hautzinger et al., 2023). Childhood maltreatment was assessed via self-report using the 28-item Childhood Trauma Questionnaire (CTQ; Bernstein et al., 2003; German version: Klinitzke et al., 2012), which evaluates five domains: emotional abuse, physical abuse, sexual abuse, emotional neglect, and physical neglect, rated on a 5-point Likert scale. Alexithymia, reflecting difficulties in identifying and expressing emotions, was assessed with the 24-item Perth Alexithymia Questionnaire (PAQ; Preece et al., 2018; German version: Kaemmerer et al., 2021), rated on a 7-point Likert scale.

Internal consistency reliability was calculated for all measures in the present sample using Cronbach’s *α* with 95% confidence intervals. The BDI-II showed excellent internal consistency [*α* = 0.95, 95%-KI (0.93, 0.97)]. The PAQ demonstrated good internal consistency [*α* = 0.88, 95%-KI (0.82, 0.93)]. For the CTQ subscales the following values were obtained: emotional abuse [*α* = 0.89, 95%-KI (0.82, 0.94)], physical abuse [*α* = 0.78, 95%-KI (0.64, 0.88)], sexual abuse [*α* = 0.66, 95%-KI (0.43, 0.81)], emotional neglect [*α* = 0.82, 95%-KI (0.71, 0.90)], and physical neglect [*α* = 0.44, 95%-KI (0.08, 0.69)].

#### 2.3.2 Ecological momentary assessment (EMA)

Using EMA via the movisensXS smartphone app, participants were prompted three times daily for 14 days to report on ER. Before the study, participants received brief training from researcher on app use and on the three ER strategies: reappraisal, suppression, and social sharing, including definitions, examples, and response options. These strategies were assessed in interpersonal situations, capturing both interpersonal (social sharing) and intrapersonal (reappraisal, suppression) regulatory processes in social interactions (e.g., through communication with close others). Random prompts were delivered between 10:00 and 18:00. EMA items were adapted and translated into German from Paul et al.’s (2023) pilot study, which investigated these ER strategies in interpersonal contexts within a non-clinical sample. At each prompt, participants recalled their most intense emotional event since the previous signal and reported on: (a) social context [close others (i.e., friends, family), non-close others (i.e., strangers), or alone], (b) emotional state (arousal, energy, valence; 11-point scales), and (c) ER strategies (reappraisal: “I thought about the situation in a different way,” social sharing: “I shared my feelings with others or talked to others about them,” expressive suppression: “I did not show my feelings”); 11-point scale from 0 = not at all to 10 = very much; see (Paul et al., 2023). Item details are provided in Supplementary Table S1.

### 2.4 Data analysis

Linear mixed models (random-intercept models) were conducted using the nlme package in R (Version 2023.09.1 + 494) (Bates et al., 2015). Measurement occasions (Level 1) were nested within individuals (Level 2), with group (depressive vs. healthy) included as a fixed effect. Three separate models were estimated with ER strategies (reappraisal, social sharing, suppression) as dependent variables. Each model included a random intercept for participants to account for repeated measurements nested within individuals and a fixed effect of group to examine between-group differences. The depressive group served as the reference level relative to the HC. The general model specification for the ER strategies was as follows:

Level 1


$$Y_{\mathrm{ij}}=\beta_{0j}+\varepsilon_{\mathrm{ij}}$$

Level 2


$$\beta_{0j}=\gamma_{00}+\gamma_{01}\text{Grou}p_{j}+u_{0j}$$

where i indexes measurement occasions, j indexes individuals, and Y represents reappraisal, social sharing, or suppression.

Model assumptions were evaluated via visual inspection of diagnostic plots (linearity, normality of residuals, homoscedasticity, and influential observations), with no substantial violations observed.

Within the depressive subsample, associations between depression severity and ER strategy use were examined using two-level linear mixed models (Level 1: measurement occasions; Level 2: individuals). Depression severity (BDI-II) was entered as a Level-2 predictor in separate models for each strategy. Given the small sample size (*N* = 35), cross-group moderation effects could not be tested. Exploratory linear mixed models examined associations between ER strategy use in close relationships and alexithymia (PAQ) as well as childhood maltreatment (CTQ), each included separately as Level-2 predictors.

Missing data were handled using maximum likelihood estimation within the mixed models, assuming data are missing at random. This approach is well established for multilevel EMA data (Enders, 2022).

*A priori* power analyses were not conducted, as the study was designed as a feasibility EMA study. A *post hoc* sensitivity analysis (Green and MacLeod, 2016) indicated that the present design was only sufficiently powered (80%) to detect relatively large standardized fixed effects (*β* ≈ 0.70–0.80). Such effects are considered large in psychological research and are uncommon in EMA studies on ER and depression, which typically report small to medium effects (*β* = 0.01–0.39) (Paul et al., 2023; Schraub et al., 2013; Pacheco et al., 2026).

## 3 Results

### 3.1 Response rates and variability in emotion regulation strategies

Considering interactions with close individuals the final sample comprised 273 assessments in the healthy group and 126 in the depressive group. Over the 14-day period, the healthy group reported 59.2% of their total social interactions with close individuals, while the group with depressive disorders reported only 34.5%, approximately half the proportion of social interactions with close individuals compared to the healthy group. Mean scores, standard deviations, and intraclass correlations between variables are presented in Supplementary Table S2. Intraclass correlations (ICCs) were calculated using a one-way random-effects model [ICC(1)] to estimate between-person variance in the EMA data. Given that much of the variability in the use of ER strategies occurs within individuals (1 − ICC, Supplementary Table S2), the need for multilevel models becomes evident, affecting both the control group (43–55%) and the depressive group (55–62%).

### 3.2 Comparing maladaptive emotion regulation strategy usage in close relationships: healthy vs. depressive participants

The Hierarchical Linear Modelling (HLM) analyses revealed no significant differences between depressive patients and HC in the intensity of ER strategies within close relationships. Reappraisal [*b* = −0.28, *t*(33) = −0.39, *p* = 0.698], suppression [*b* = 0.07, *t*(33) = 0.09, *p* = 0.922], and social sharing [*b* = 0.16, *t*(33) = 0.19, *p* = 0.850] did not differ between groups (see Table 1). Group-wise descriptive statistics for ER strategy intensity are provided in Supplementary Table S3.

**Table 1:** Differences between healthy and depressed participants in the use of emotion regulation strategies in close interpersonal contexts.

Predictors	Reappraisal				Suppression				Social sharing			
	*b*	SE *b*	95% CI	*p*	*b*	SE *b*	95% CI	*p*	*b*	SE *b*	95% CI	*p*
(Intercept)	3.54	0.53	[2.51, 4.57]	**<0.001**	4.48	0.53	[3.43, 5.53]	**<0.001**	4.26	0.63	[3.03, 5.49]	**<0.001**
Group (healthy)	−0.28	0.72	[−1.73, 1.17]	0.698	0.07	0.72	[−1.40, 1.54]	0.922	0.16	0.86	[−1.57, 1.90]	0.850
Marginal *R*^2^	0.004				0.000				0.001			

Group = fixed effect. Group of healthy participants *n* = 18 and 273 observations, group of depressed participants *n* = 17 and 126 observations. *b* = estimated fixed effect. SE *b* = standard error of the estimate. 95% CI, 95% confidence interval. Values in bold indicate statistical significance at *p* < 0.05, *p* < 0.01, and *p* < 0.001.

### 3.3 Correlation of depressive symptom severity with emotion regulation strategy usage in close relationships

We additionally examined whether baseline depressive symptom severity predicted the use of ER strategies in close relationships among individuals with depressive disorders. No significant associations were observed for any of the three strategies [reappraisal: *b* = −0.01, *t*(15) = −0.23, *p* = 0.822; suppression: *b* = 0.03, *t*(15) = 0.67, *p* = 0.514; social sharing: *b* = −0.08, *t*(15) = −1.61, *p* = 0.128; see Table 2]. Overall, depressive symptom severity did not significantly predict the intensity of ER strategies in close interpersonal contexts.

**Table 2:** Associations between the severity of depressive symptoms and the intensity of emotion regulation strategies in close interpersonal contexts.

Predictors	Reappraisal				Suppression				Social sharing			
	*b*	SE *b*	95% CI	*p*	*b*	SE *b*	95% CI	*p*	*b*	SE *b*	95% CI	*p*
(Intercept)	3.78	1.22	[1.38, 6.17]	**0.002**	3.71	1.26	[1.24, 6.19]	**0.004**	6.29	1.39	[3.57, 9.02]	**<0.001**
BDI	−0.01	0.04	[−0.10, 0.08]	0.822	0.03	0.04	[−0.06, 0.12]	0.514	−0.08	0.05	[−0.18, 0.02]	0.128
Marginal *R*^2^	0.003				0.022				0.129			

*n* = 17 depressive patients and 126 observations. BDI = severity of depressive symptoms. b = estimated fixed effect of depression severity on the intensity of ER strategies in close interpersonal contexts. SE *b* = standard error of the estimate. 95% CI, 95% confidence interval. Values in bold indicate statistical significance at *p* < 0.05, *p* < 0.01, and *p* < 0.001.

### 3.4 Exploratory analyses

First, group differences in alexithymia severity were examined across the full sample (healthy vs. depressive). Participants with depressive disorders reported significantly higher alexithymia scores compared to HC, *t*(33) = 2.40, *p* = 0.02, Cohen’s *d* = 0.81 (see Supplementary Figure 1).

Subsequently, *post-hoc* analyses were conducted to explore associations between alexithymia severity, childhood maltreatment (based on total scores from the respective questionnaires), and the use of ER strategies within the depressive group only (*n* = 17). Linear mixed models were applied separately for each strategy, focusing on their use in close interpersonal contexts.

Results showed a significant negative association between alexithymia severity (PAQ scores) and social sharing among individuals with depression [*b* = −0.04, *t*(15) = −2.67, *p* = 0.017; Supplementary Table S4]. No significant associations were found for reappraisal or suppression (all *p*s ≥ 0.68, see Supplementary Table S4).

*Post-hoc* analyses further examined associations between childhood maltreatment (CTQ) and ER strategies in the depressed group using three linear mixed models (one per strategy). Cognitive reappraisal was significantly associated with all five facets of childhood maltreatment (Table 3). Emotional abuse [*b* = 0.31, *t*(11) = 5.27, *p* < 0.001], sexual abuse [*b* = 1.07, *t*(11) = 5.05, *p* < 0.001], and physical neglect [*b* = 0.39, *t*(11) = 3.75, *p* = 0.003] were positively associated with greater reappraisal intensity. By contrast, physical abuse [*b* = −0.21, *t*(11) = −2.34, *p* = 0.039] and emotional neglect [*b* = −0.24, *t*(11) = −2.64, *p* = 0.023] were negatively associated with reappraisal intensity. These findings suggest that depressive individuals who experienced more emotional or sexual abuse or physical neglect in childhood may engage in greater reappraisal, whereas more severe physical abuse or emotional neglect is associated with less intensive reappraisal use. No significant associations were found for suppression or social sharing (all *p*s > 0.124; see Supplementary Table S5).

**Table 3:** Associations between childhood maltreatment subdomains and reappraisal intensity in close interpersonal contexts.

Predictors	Reappraisal		
	*b*	95% CI	*p*
(Intercept)	−4.91	[−6.93, −2.88]	**<0.001**
Emotional abuse	0.31	[0.18, 0.44]	**<0.001**
Physical abuse	−0.21	[−0.41, −0.02]	**0.039**
Sexual abuse	1.07	[0.61, 1.52]	**<0.001**
Emotional neglect	−0.24	[−0.43, −0.04]	**0.023**
Physical neglect	0.39	[0.17, 0.61]	**0.003**
Marginal *R*^2^	0.418		

*n* = 17 depressive patients and 126 observations. *b* = estimated fixed effect of childhood maltreatment subdomains on the intensity of cognitive reappraisal in close interpersonal contexts. 95% CI, 95% confidence interval. Values in bold indicate statistical significance at *p* < 0.05, *p* < 0.01, and *p* < 0.001.

## 4 Discussion

Our findings did not reveal significant differences in the intensity of ER strategy use between depressive and healthy participants in interactions with close others. This is in contrast with meta-analytic evidence from Aldao et al. (2010), who found that depressed individuals generally use more maladaptive strategies (e.g., suppression, rumination) and less adaptive strategies (e.g., reappraisal) than HC. A possible explanation for these heterogenous outcomes may be Aldao et al. (2010) not accounting for social context, whereas our study focused specifically on close interpersonal relationships. Supporting this interpretation, neurological research shows that attachment-related cues, such as the familiar faces of close others, activate semantic networks involved in voluntary ER, including cognitive reappraisal (Gobbini et al., 2004; Buhle et al., 2014). Close relationships may therefore naturally increase the likelihood of attempting to reinterpret interpersonal situations. Compared to HC, depressed individuals tend to rely more rigidly on cue-driven, stimulus-triggered reappraisal rather than on flexible cost–benefit evaluations (Choi et al., 2025; Haines et al., 2016), a pattern associated with poorer strategy-situation fit (Haines et al., 2016). Together, these findings provide a theoretical rationale for expecting heightened, but potentially inflexible, reappraisal use in close interpersonal contexts among individuals with depression. Given the focus on close interpersonal interactions, the lack of group differences may reflect the “dilution” effect of social connectedness (Marroquín and Nolen-Hoeksema, 2015), where supportive partners or relational dynamics reduce the influence of both maladaptive and adaptive strategies. In this sense, proximal social context may buffer the impact of maladaptive ER strategies while also reducing the relative benefit of adaptive strategies, which could therefore have influenced results of this study. However, additional exploratory analyses including broader social contexts (non-close interactions and being alone) did not reveal significant group-by-context interactions, suggesting that the absence of group differences cannot be fully explained by the restriction to close relationships. This interpretation therefore remains speculative, highlighting the need for further research on how relationship quality, such as intimacy, trust, and perceived support, moderates ER in depressive disorders, as explored by Marroquín and Nolen-Hoeksema (2015). Overall, ER differences observed in broader contexts may be less pronounced in supportive close relationships. Given the sample size, the study was not sufficiently powered to examine group as a moderator of associations between ER strategies and depressive symptoms. Future studies should investigate whether these relationships differ between individuals with and without depressive disorders.

Exploratory analyses revealed that individuals with depression exhibited significantly higher levels of alexithymia compared to HC (Supplementary Figure 1). Alexithymia was positively associated with depression severity (Supplementary Table S6), consistent with prior findings (Sagar et al., 2021). Among depressed individuals, alexithymia was significantly negatively correlated with social sharing (Supplementary Table S4). This aligns with evidence that alexithymia is linked to reduced use of adaptive strategies and increased reliance on maladaptive ones (Preece et al., 2023). Given the social function of emotions in communicating internal states and eliciting responses (Keltner and Gross, 1999), social sharing can be understood as an interpersonal strategy that relies on emotional expression to obtain support. Difficulties in these processes, as seen in alexithymia, may therefore hinder social sharing, particularly in depression and among individuals with adverse childhood experiences, who often struggle with emotional communication (Honkalampi et al., 2020). These findings suggest a potential pathway whereby childhood maltreatment contributes to elevated alexithymia (Honkalampi et al., 2020; Khan and Jaffee, 2022), which may impair interpersonal ER (Nowicki, 2025), and increase vulnerability to depressive symptoms (Marroquín, 2011). Future research should test this sequence empirically to clarify the interplay between early adversity, alexithymia, and interpersonal ER in depression.

In addition, post-hoc analyses revealed mixed associations between reappraisal and childhood maltreatment. Emotional abuse, sexual abuse, and physical neglect were associated with higher reappraisal use, whereas physical abuse and emotional neglect were associated with lower reappraisal use. Given the small sample size and multiple comparisons without statistical adjustment, these findings should be considered preliminary and interpreted with caution. Moreover, the low internal consistency of the physical neglect subscale in the present sample further limits the interpretation and stability of its association with reappraisal. However, prior evidence suggests that childhood maltreatment may limit the capacity to implement reappraisal effectively (Armbruster-Genç et al., 2025), indicating that increased reappraisal attempts might not achieve the desired regulation of negative affect and could be linked to tendencies such as emotional withdrawal. This process should be interpreted with caution due to limited empirical evidence supporting such mechanisms. The observed mixed pattern also complicates clear conclusions regarding the role of reappraisal in close interpersonal contexts. In light of Goerigk et al. (2024), childhood maltreatment may be most informative when examined using cluster-based approaches. However, this was not feasible here due to the small sample size, which can produce unstable clustering solutions (Nylund-Gibson and Choi, 2018; Tein et al., 2013). Traditional CTQ subscales may oversimplify the complexity of early adversity and its links to ER and interpersonal functioning. Future research should therefore apply cluster-based methods to better capture maltreatment profiles and their implications for depression and ER.

Although reappraisal is typically considered an adaptive strategy, reliance on self-report may introduce bias (Podsakoff et al., 2003), limiting conclusions about its effective implementation. Accordingly, reported reappraisal may reflect maladaptive cognitive processes such as minimization (downplaying the importance of negative events) or avoidance (trying to escape negative feelings). Future research should clarify the conditions under which reappraisal is beneficial and develop more objective measures of its use.

Regarding the hypothesis of elevated suppression in depressed individuals, several factors may explain the lack of significant group differences. First, item wording for suppression may have led to misunderstandings (see Supplementary Table S1). Second, suppression may be emotion-specific, and averaging across emotions could have reduced sensitivity. Third, responses may reflect habitual rather than situation-specific tendencies.

Depressive participants also reported substantially fewer close interpersonal interactions than HC (34.5% vs. 59.2%). This imbalance may have reduced statistical power to detect between-group differences. Moreover, the unequal frequency of close interactions may have resulted in systematic differences in the interpersonal contexts sampled across groups, thereby limiting the comparability of the observed experiences. Thus, the lack of significant group differences should be interpreted cautiously and may not necessarily indicate true regulatory equivalence.

### 4.1 Limitations

As the sensitivity analysis revealed, the present study was limited in statistical power and therefore primarily sensitive to relatively large between-group effects. Furthermore, the study relied solely on self-report measures of ER strategy use, which may not accurately reflect actual behavior or strategy effectiveness. The EMA single-item measures in the present study were adopted from previously published EMA protocols (Paul et al., 2023) and have been used in subsequent research (Mueller et al., 2024). Although similar single-item EMA measures have shown evidence of reliability and validity (Pruessner et al., 2023), the specific measures used in the present study have not been formally validated. Their use may therefore not fully capture the complexity of each ER strategy and may limit the generalizability of the findings. Finally, a single baseline assessment of depressive symptoms may not capture temporal fluctuations.

Despite these limitations, the findings may tentatively indicate that individuals with depression, particularly those with higher symptom severity, could engage less in social interactions, which might be relevant for ER use in close relationships. Future studies should employ larger samples, repeatedly assess depressive symptoms, and consider momentary affect to further clarify mechanisms of interpersonal ER in depression.

## 5 Conclusion

In summary, no distinct maladaptive ER pattern emerged between depressed and healthy participants in close relationships. Given the small sample size, smaller or moderate group differences may have gone undetected. Cognitive reappraisal may nevertheless be influenced by specific adverse childhood experiences in depressed individuals, which may lead to either increased or decreased use of reappraisal depending on the type of maltreatment. This suggests that, despite being generally adaptive, reappraisal may be less effective or more complex in close interpersonal interactions among those with depressive disorders. Additionally, the study found that higher alexithymia was associated with reduced social sharing among individuals with depressive disorders, highlighting the importance of considering alexithymia in understanding and addressing interpersonal ER difficulties in depressive disorders. Overall, the present findings provide valuable insights and directions for future research on ER and depression. While some results were conflicting, they highlight important associations and mechanisms that warrant further investigation, particularly regarding the influence of social context (i.e., close interpersonal contexts), comorbid alexithymia, and histories of childhood maltreatment on ER patterns in depressive disorders.

## Data Availability

The raw data supporting the conclusions of this article will be made available by the authors, without undue reservation.
