# Supplementary material for: Interpersonal emotion regulation in daily life: a comparison of individuals with and without depressive disorders
Source: Front Psychol. 2026 Sep 30;17:1773706. doi: 10.3389/fpsyg.2026.1773706 (PMC13661391; doi:10.3389/fpsyg.2026.1773706)
Supplement: Supplementary file 1 [file supplementary_file_1.docx]

Supplementary Material

# Supplementary Figures and Tables

## 1. Supplementary Tables

**Table S1**

**Surveys Used in this Study. German Translation of the Original Items in the Preliminary Study by Paul et al. (2023)**

|  | **Original Items** | **Translated Items** |
| --- | --- | --- |
| **Social Context** | Who was present in the situation? | Wer war in dieser Situation anwesend? |
|  | Friends/ family/ romantic partner | Freunde/ Familie/ Intimpartner:in |
|  | Strangers/ acquaintances/ colleagues | Fremde / Bekannte / Kolleg*innen |
|  | Being alone | Alleinsein |
| **Emotional State** | How did you feel in the situation? (11-point Likert scale) | Wie haben Sie sich in dieser Situation gefühlt? (11-Punkt Likert-Skala) |
| Arousal | 0 = relaxed/calm, 10 = tense/agitated | 0 = ruhig/entspannt, 10 = unruhig/angespannt |
| Energy | 0 = tired/without energy, 10 = awake/full of energy | 0 = müde/energielos, 10 = wach/ energiegeladen |
| Valence | 0 = unwell/discontent, 10 = well/content | 0 = unwohl/unzufrieden, 10 = wohl/zufrieden |
| **Emotion Regulation** | How did you deal with your feelings in that situation?  Rating on a 11-point Likert scale (0 = not at all to 10 = very much) | Wie sind Sie in dieser Situation mit Ihren Gefühlen umgegangen?  Bewertung auf einer 11-Punkt Likert-Skala (0 = gar nicht bis 10 = äußerst) |
| Reappraisal | I thought about the situation in a different way | Ich habe die Situation anders betrachtet |
| Social sharing | I shared my feelings with others or talked to others about them | Ich habe meine Gefühle mit anderen geteilt oder mit anderen darüber gesprochen |
| Suppression | I did not show my feelings | Ich habe meine Gefühle nicht gezeigt |

**Table S2**

**Person-level Means (*M*), Standard Deviation (*SD*), and Intraclass Correlation Coefficient (ICC)**

|  |  | **Group of healthy subjects** | | | | **Group of depressive subjects** | | | |
| --- | --- | --- | --- | --- | --- | --- | --- | --- | --- |
| Variables | | *M* | *SD*_between_ (*n*=18 participants) | *SD*_within_ (*n*=273 assessments) | ICC | *M* | *SD*_between_ (*n*=17 participants) | *SD*_within_ (*n*=126 assessments) | ICC |
| Emotion Regulation Strategies* | | | | | | | | | |
| 1. | Reappraisal | 3.76 | 2.18 | 2.01 | 0.54 | 3.29 | 1.77 | 2.15 | 0.40 |
| 2. | Suppression | 4.83 | 2.10 | 2.30 | 0.45 | 4.57 | 1.86 | 2.36 | 0.38 |
| 3. | Social sharing | 4.72 | 2.60 | 2.23 | 0.57 | 4.22 | 2.21 | 2.44 | 0.45 |

Note. ICC = intraclass correlation coefficient, reflecting the proportion of variance in emotion regulation strategy use that lies between individuals. *Emotion regulation strategies are considered only in close interpersonal contexts and can vary on an 11-point Likert scale (from 0 = not at all to 10 = very much).

**Table S3**

**Descriptive Statistics of Emotion Regulation Strategy Intensity in the Depressive and Healthy Control Groups**

| Groups | **Reappraisal**_*M* (*SD*) | **Suppression**_*M* (*SD*) | **Social sharing**_*M* (*SD*) |
| --- | --- | --- | --- |
| **Depressive** | 3.66 (1.98) | 4.43 (2.01) | 4.33 (2.35) |
| **Healthy Control** | 3.18 (2.27) | 4.48 (2.27) | 4.41 (2.73) |

Note. Depressive group *n* = 17, Healthy control group *n* = 18. *M* = mean; *SD* = standard deviation.

**Table S4**

**Associations Between Alexithymia Severity and the Intensity of Emotion Regulation Strategies in Close Interpersonal Contexts**

|  | **Reappraisal** | | | | **Suppression** | | | | **Social sharing** | | | |
| --- | --- | --- | --- | --- | --- | --- | --- | --- | --- | --- | --- | --- |
| Predictors | *b* | SE *b* | 95% CI | *p* | *b* | SE *b* | 95% CI | *p* | *b* | SE *b* | 95% CI | *p* |
| (Intercept) | 2.96 | 1.45 | [0.12, 5.80] | **0.043** | 4.18 | 1.53 | [1.18, 7.18] | **0.007** | 7.85 | 1.44 | [5.02, 10.68] | **<0.001** |
| **PAQ** | 0.01 | 0.02 | [-0.03, 0.04] | 0.687 | 0.00 | 0.02 | [-0.03, 0.04] | 0.835 | -0.04 | 0.02 | [-0.07,-0.01] | **0.017** |

Note. *n* = 17 depressive patients and 126 observations. PAQ (Perth Alexithymia Questionnaire) measures the severity of alexithymia. *b* = estimated fixed effect of alexithymia severity on the intensity of emotion regulation strategy use in close interpersonal contexts. 95% CI = 95% confidence interval.

**Table S5**

**Correlations Between Childhood Maltreatment Subdomains and the Intensity of Suppression and Social Sharing in Close Interpersonal Contexts**

|  | **Suppression** | | | **Social sharing** | | |
| --- | --- | --- | --- | --- | --- | --- |
| Predictors | *b* | 95% CI | *p* | *b* | 95% CI | *p* |
| (Intercept) | 1.12 | [-3.88, 6.12] | 0.666 | 2.10 | [-4.22, 8.43] | 0.521 |
| Emotional Abuse | 0.01 | [-0.29, 0.30] | 0.963 | 0.11 | [-0.26, 0.48] | 0.541 |
| Physical Abuse | -0.13 | [-0.58, 0.32] | 0.549 | -0.03 | [-0.60, 0.53] | 0.902 |
| Sexual Abuse | 0.38 | [-0.69, 1.44] | 0.464 | 0.08 | [-1.25, 1.42] | 0.895 |
| Emotional Neglect | -0.05 | [-0.45, 0.34] | 0.785 | -0.18 | [-0.67, 0.31] | 0.438 |
| Physical Neglect | 0.33 | [-0.10, 0.76] | 0.124 | 0.34 | [-0.18, 0.87] | 0.190 |

Note. *n* = 17 depressive patients and 126 observations. *b* = estimated fixed effect of childhood maltreatment subdomains on the intensity of suppression and social sharing in close interpersonal contexts. 95% CI = 95% confidence interval.

**Table S6**

**Correlation Between Depressive Symptom Severity and Alexithymia**

|  | **Alexithymia** | | | |
| --- | --- | --- | --- | --- |
| Predictors | *b* | SE *b* | 95% CI | *p* |
| (Intercept) | 60.78 | 7.16 | [46.22, 75.34] | **<0.001** |
| **Depression** | 1.27 | 0.35 | [0.56, 1.97] | **0.001** |
| Observations | 35  0.287/0.266 | | | |
| R^2^/ R^2^ adjusted |  |  |  |  |

Note. *n* = 35 participants. *b* = estimated fixed effect of depressive symptom severity on alexithymia in the full sample. 95% CI = 95% confidence interval.

## 2. Supplementary Figures

**Supplementary Figure 1. Group Differences in Alexithymia: Healthy Controls vs. Depressive Participants**

Note. Two-Sample t-Test: *t*(33) = 2.40, *p* = .02, Cohen’s *d* = 0.81. Healthy participants: *M* = 68.44 (*SD* = 27.67); depressive participants: *M* = 93.17 (*SD* = 33.0).
